# Predictive modelling of Alzheimer's Disease progression using Machine Learning Algorithms

**DOI:** 10.1002/alz70858_096925

**Published:** 2025-12-24

**Authors:** Gideon S Alex, Ibeachu Chinagorom, NeurodataHub

**Affiliations:** ^1^ University of Port‐Harcourt Nigeria, Port‐Harcourt, Rivers, Nigeria; ^2^ University of Port Harcourt, Port Harcourt, Rivers State, Nigeria

## Abstract

**Background:**

Alzheimer's disease is the most common form of dementia, a significant contributor to the global burden of disability and death globally. Early diagnosis and accurate prediction of disease progression are crucial for effective management and treatment plan.

**Method:**

We utilized publicly available neuroimaging dataset comprising 2D axial MRI scans from three classes: Alzheimer's disease, Cognitive Impaired and Cognitive normal. Preprocessing of data was performed using OpenCV (Open source computer vision library). The models employed were ResNet50 and DenseNet121. Model performance was evaluated using accuracy, loss, AUC and confusion matrices

**Result:**

Our results showed that both ResNet50 and DenseNet121 achieved exceptional accuracy of 99.8% and 100% respectivly. Confusion matrices of both models showed excellent performance in classifying images with AUC value exceeding 0.99.

**Conclusion:**

The study demonstrates the potential of machine learning in predicting the progression of Alzheimer's disease. Our finding suggest that machine learning models can help clinicians and healthcare professionals

